# Exploring the therapeutic potential of puerarin on intervertebral disc degeneration by regulating apoptosis of nucleus pulposus cells

**DOI:** 10.1002/jsp2.70020

**Published:** 2024-12-11

**Authors:** Xiaoqiang Wang, Chao Song, Daqian Zhou, Yongliang Mei, Weiye Cai, Rui Chen, Jiale Lv, Houyin Shi, Zongchao Liu

**Affiliations:** ^1^ Department of Orthopedics and Traumatology, The Affiliated Traditional Chinese Medicine Hospital Southwest Medical University Luzhou China; ^2^ Department of Orthopedics Luzhou China

**Keywords:** apoptosis, bioinformatics, intervertebral disc degeneration, nucleus pulposus cells, puerarin

## Abstract

Intervertebral disc degeneration (IVDD) stands as a prevalent chronic orthopedic ailment, profoundly impacting patients' well‐being due to incapacitating low back pain. Studies have highlighted a close correlation between IVDD and the programmed cell death of nucleus pulposus (NP) cells orchestrated by interleukin‐1 beta (IL‐1β), tumor necrosis factor‐alpha (TNF‐α), and caspase‐3 (CASP3). Puerarin, renowned for its anti‐inflammatory attributes and its influence on IL‐1β and TNF‐α, emerges as a promising candidate for IVDD treatment. However, the precise mechanism by which it regulates apoptosis via these pathways remains ambiguous. This investigation utilizes bioinformatics to unveil the molecular intricacies of puerarin‐mediated apoptosis regulation in IVDD, substantiated by preliminary in vitro experiments. Analysis exposes aberrant expression of pivotal apoptosis‐associated proteins (IL‐1β, TNF‐α, CASP3, CASP8, and BCL2) in IVDD patients, with network pharmacology indicating puerarin's potential efficacy in IVDD treatment by modulating apoptosis and cellular senescence pathways. Further experiments elucidate puerarin's capacity to stimulate NP cell proliferation while inhibiting apoptosis, potentially contributing to IVDD mitigation. Western blot and PCR outcomes reveal escalated expression of apoptosis‐related proteins (IL‐1β, TNF‐α, and CASP3) in lipopolysaccharide‐treated NPCs, ameliorated by puerarin intervention. Molecular docking simulations demonstrate favorable binding properties of puerarin with apoptotic proteins, while flow cytometry analysis indicates its ability to diminish NPC apoptosis. These discoveries imply that puerarin might alleviate NPC apoptosis by modulating key targets, thereby potentially ameliorating IVDD. In summary, this study unveils the intrinsic mechanism of puerarin in regulating NPC apoptosis to alleviate IVDD, underscoring its therapeutic promise.

## Graphical abstract

Network pharmacology and bioinformatics analyses suggest that puerarin may mitigate intervertebral disc degeneration (IVDD) by inhibiting nucleus pulposus (NP) cell apoptosis. This hypothesis was validated through in vitro experiments, which demonstrated that puerarin significantly reduces apoptosis in NPCs.

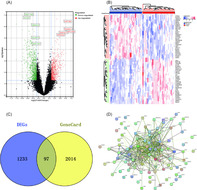

## 1 INTRODUCTION

Intervertebral disc degeneration (IVDD) stands as a prevalent chronic ailment in orthopedics, commonly afflicting middle‐aged and elderly individuals, with low back pain (LBP) constituting its primary clinical manifestation.^1^, ^2^ LBP, a ubiquitous symptom of spinal diseases, poses a significant global public health concern, boasting a lifetime prevalence as high as 84% and costing the United States an estimated US$10 billion annually.^3^ Comprising upper and lower cartilage endplates, the surrounding annulus fibrosus, and the central nucleus pulposus (NP), the intervertebral disc plays a pivotal role in spinal weight‐bearing and mobility.^4^ Presently, clinical strategies for alleviating IVDD predominantly entail conservative measures and surgical interventions, with surgery often considered if conservative approaches prove ineffective in pain relief. Nevertheless, these interventions primarily target symptom management rather than addressing the root cause.^5^, ^6^ Prior investigations by our research team have delineated IVDD's pathogenesis, highlighting associations with cellular aging, immune dysregulation, genetic predispositions, apoptosis, pyroptosis, and heightened release of inflammatory cytokines.^7^, ^8^, ^9^, ^10^, ^11^ Hence, a comprehensive understanding of IVDD's pathophysiology is imperative for devising more effective therapeutic modalities.

The intervertebral disc typically exists within a relatively enclosed environment, resulting in the NP tissue lacking direct blood supply.^12^ Nucleus pulposus cells (NPCs) inhabit a hypoxic and nutrient‐deficient microenvironment.^9^, ^13^ This unique disc structure renders NPCs highly sensitive to microenvironmental changes, particularly the accumulation of inflammatory factors.^14^ Recent research has underscored the pivotal role of inflammatory response in the pathogenesis and progression of NP tissue degeneration. Elevated levels of inflammatory cytokines such as tumor necrosis factor‐α (TNF‐α) and interleukin‐1β (IL‐1β) disrupt the balance between extracellular matrix synthesis and degradation, thereby promoting NPC death.^15^, ^16^ Consequently, preventing or mitigating NPC death is deemed crucial in averting IVDD and restoring intervertebral disc functionality.

Cell death mechanisms encompass accidental cell death (ACD) and regulated cell death (RCD), the latter occurring under physiological conditions and often referred to as programmed cell death (programmed cell death, PCD).^17^, ^18^, ^19^ Building upon our previous research, we investigated the mechanism of apoptosis in IVDD. Apoptosis, a primary form of cell death, serves critical roles in tissue and organ development, growth regulation, and the clearance of autoreactive immune cells, thereby preserving multicellular organism homeostasis. Conversely, dysregulated apoptosis is closely linked to various diseases, including tumors, autoimmune disorders, and degenerative condition.^20^, ^21^ Factors contributing to IVDD encompass oxidative stress, aging, nutritional deficiencies, genetic predispositions, and mechanical loading.^7^ Early stages of IVDD coincide with increased infiltration of inflammatory factors, such as IL‐1β, tumor necrosis factor, and IL‐18.^8^ These inflammatory mediators, along with pyroptosis triggered by NLRP3 inflammasome activation, not only diminish the quantity and quality of NPCs but also induce cellular oxidative stress damage.^7^ Recent research has highlighted widespread activation of the NLRP3 inflammasome in IVDD, mediating the production of various inflammatory cytokines and further exacerbating the disease.^22^ Moreover, our investigation revealed heightened expression of key apoptosis proteins, including CASP and BCL2, in human intervertebral disc NPCs. This observation suggests a potential role for apoptosis in IVDD pathogenesis.^11^

In traditional Chinese medicine, IVDD falls under the category of “Bi syndrome” and “lumbar paralysis.” Pueraria lobata is regarded in traditional Chinese medicine as one of the effective remedies for managing “Bi syndrome.”^23^ Modern pharmacological studies suggest that Pueraria lobata may offer a range of potential benefits, including delaying IVDD, enhancing bone metabolism, inhibiting inflammatory mediator activity, improving microcirculation, and exerting antioxidant and vasodilatory effects. These findings indicate the possibility of its use in clinical practice, though further research is required to confirm its efficacy.^24^ Research has spotlighted puerarin, the principal compound in Pueraria lobata, for its role in decelerating IVDD progression. Specifically, studies indicate that puerarin's ability to delay cervical IVDD may be linked to its modulation of phospholipase A2 activity, prostaglandin E2 levels, downregulation of Fas expression, and upregulation of Bcl‐2 expression in intervertebral disc tissue. However, whether puerarin can ameliorate the inflammatory response of NPCs by regulating apoptosis remains unclear. Thus, elucidating the apoptotic molecular mechanism through which puerarin regulates NPC death holds significant implications for IVDD treatment.^25^, ^26^, ^27^

To identify pivotal genes regulating apoptosis and inflammation in IVDD patients, we scrutinized the GSE56081, GSE150408, GSE124272, and GSE153761 datasets in the Gene Expression Omnibus (GEO) database, as well as the Genecard database,^28^, ^29^, ^30^ to gather information on IVDD disease‐associated hub genes. Our analysis yielded 31 hub genes closely linked to IVDD, apoptosis, and inflammation. Additionally, leveraging the TCMSP Traditional Chinese Medicine Database, we conducted an extensive examination of puerarin's molecular mechanism in IVDD treatment. To validate our findings, we conducted a series of in vitro experiments, ultimately confirming that regulating NPC apoptosis is pivotal in IVDD treatment, with puerarin effectively targeting this signaling pathway to ameliorate IVDD. We posit that this study lays a robust theoretical foundation for IVDD treatment and facilitates the clinical application of traditional Chinese medicine.

## 2 MATERIALS AND METHODS

### 2.1 Bioinformatics data mining

#### 2.1.1 Microarray data source (MDS) and differential gene analysis (DGA)

Gene microarray data for IVDD were retrieved from the NCBI GEO public database. Specifically, data from GSE56081 included 5 IVDD samples and 5 normal controls, data from GSE150408 comprised 17 IVDD samples and 17 normal controls, data from GSE124272 consisted of 8 IVDD samples and 8 normal controls, and data from GSE153761 contained 3 IVDD samples and 3 normal controls (Supplementary Table S1).^31^ The normal control samples were sourced from cadaveric donors or patients with vertebral fractures. Sangerbox 3.0 was utilized to convert the microarray matrix data into gene expression matrix data by gene name. Subsequently, a data fusion and batch effect removal process was performed using the “Multi‐data aggregation and batch effect removal tool” to obtain valid raw data after batch effect elimination. DGA was conducted using the “limma” tool on the valid raw data to identify differentially regulated genes. Volcano plots were generated to visualize differences in gene expression levels between normal and IVDD samples.^32^ Parameters were set as follows: fold change ≥1.2, *p* < 0.05, with the Euclidean distance calculation method and complete clustering method employed to construct differential gene heat maps.^33^ The Genecards disease database (https://www.genecards.org/) was queried with the keyword “intervertebral disc degeneration” to identify target proteins involved in the disease.^31^ The collected disease target data were then intersected with the differentially regulated genes to identify disease‐regulated differentially regulated genes associated with IVDD. Protein–protein interaction (PPI) network analysis of disease‐regulated differential genes for IVDD was conducted using the STRING database (http://string-db.org/), limited to “Homo sapiens” with a confidence level >0.4.^34^

#### 2.1.2 Weighted correlation network analysis

We utilized the weighted gene co‐expression network analysis (WGCNA) software package within Sangerbox 3.0 (implemented in R software) to construct a gene co‐expression network from the effective matrix raw data.^35^ Initially, we pruned the clustering tree by eliminating the top 50% of genes with the smallest median absolute deviation. Subsequently, we computed the correlation coefficient between each pair of genes and constructed a similarity matrix. To ensure the creation of a scale‐free network, we selected an appropriate soft threshold to convert the similarity matrix into an adjacency matrix. Next, we generated a topological overlap matrix to measure the average network connectivity of each gene. Using the parameters of the block‐dimensional module function, we employed a dynamic tree cutting method to group genes with similar expression profiles into distinct modules. Each module was visually represented with a unique color, with gray modules indicating genes unassigned to any module. The gene expression profile of each module was characterized by its first principal component, termed the module eigengene (ME), which served to assess the association between modules and phenotypes. The module showing the highest absolute value of the correlation coefficient was identified as the key module for further analysis. Furthermore, we calculated module membership (MM), representing the correlation coefficient between a gene's expression value and the ME of a module, indicating the gene‐module correlation. Gene significance (GS) was determined as the correlation coefficient between a gene's expression value and a phenotype, indicating the gene‐phenotype correlation. To extract hub genes, we set the MM threshold to 0.8, the GS threshold to 0.1, and the weight threshold to 0.1.

#### 2.1.3 Hub gene screening

Through WGCNA's Hub module gene screening, we identified the IVDD module gene set. We further intersected this module gene set with Genecards disease targets to obtain module hub genes with high credibility. Additionally, we combined the disease‐regulated differential genes and module hub genes of IVDD to eliminate duplicate targets and derive the final hub gene dataset effectively regulating IVDD. Subsequently, we employed the CytoHubba algorithm within Cytoscape 3.9.1 software to screen the identified key hub genes, resulting in the selection of the top 30 hub genes. Lastly, we conducted KEGG signaling pathway enrichment analysis based on the hub genes that effectively regulate IVDD to unveil disease‐related signaling pathways involved in the IVDD process.

#### 2.1.4 Molecular mechanism of puerarin in treating IVDD

We utilized the Traditional Chinese Medicine Systems Pharmacology Database (TCMSP) analysis platform (https://tcmspw.com/tcmsp.php) to gather active ingredients and targets of puerarin. Puerarin data were filtered based on criteria including oral bioavailability (OB) ≥ 30%, drug likeness (DL) ≥ 0.18, and adherence to the absorption, distribution, metabolism, and excretion (ADME) procedure.

The dataset of puerarin's drug targets was then intersected with the hub genes effectively regulating IVDD to identify potential therapeutic targets. Subsequently, enrichment analysis was conducted on the intersecting therapeutic targets to unveil expression outcomes of the KEGG signaling pathway. Finally, Cytoscape 3.9.1 was employed to construct the “puerarin‐hub gene‐signaling pathway‐IVDD” regulatory network diagram. Through this network diagram, we identified IL‐1β, TNF‐a, CASP 3, CASP8, and BCL2 as the key targets of puerarin in IVDD treatment. To further explore the expression differences of these targets in IVDD, differential box plots were utilized to depict the expression levels of these genes.

#### 2.1.5 Molecular docking

To confirm puerarin's binding activity with its key target proteins, we utilized Autodock Vina (version 1.2.2) to assess the binding energy. Subsequently, PyMol (version 2.5.5) was used for the visualization of the obtained results.

### 2.2 In vitro experimental verification

#### 2.2.1 Experimental cells and reagent selection

Healthy human primary NPCs (HUM‐iCell‐s012) were procured from iCell. Puerarin (L118716), with a purity of ≥94%, was obtained from Aladdin Reagent Company in China, and lipopolysaccharide (LPS, ST1470) was purchased from Beyotime Company. The MEM/F12 culture medium (SH30023.01) was sourced from HyClone Company in the United States, while fetal bovine serum (FBS, A5256701) was obtained from Gibco Company in the United States. IL‐1β (66737‐1‐Ig), TNF‐a (60291‐1‐Ig), CASP3 (19677‐1‐AP), CASP8 (66093‐1‐Ig), and BCL2 (60178‐1‐Ig) antibodies were purchased from Proteintech Company, along with corresponding primers procured from GeneCopoeia Company. Furthermore, the CCK‐8 kit (CA1210) and apoptosis detection kit (CA1020) were acquired from Beijing Solarbio Technology Co., Ltd.

#### 2.2.2 Experimental methods

##### Preparation of puerarin solution

10 mg of puerarin should be dissolved in 240.165 μL of DMSO to create a 100 mmol/L Pue stock solution. This stock solution should then be aliquoted and stored in a −20°C refrigerator for future use. For experimental use, the puerarin stock solution should be diluted with MEM/F12 medium containing 10% fetal bovine serum (FBS) to obtain the desired concentrations. The following concentrations can be prepared: 0.01, 0.1, 1, 10, and 100 μmol/L. These diluted solutions can be used for further experimentation.

##### Cell culture

For the culture of NPCs, we followed the recommendations of Basatvat et al.^36^ Human primary NPCs were seeded into MEM/F12 (Gibco, A4192001) culture medium supplemented with 10% fetal bovine serum (FBS, Gibco, A5256701). Subsequently, the cells were incubated in a standard cell culture incubator at 37°C with 5% CO_2_ for static culture. The growth of the cells was regularly monitored under a microscope. Upon reaching approximately 80% confluence and optimal fusion, the cells were passaged once. For experimentation, cells from the 3rd and 4th passages were utilized.

##### CCK‐8 detection

The NPCs were cultured in a 96‐well plate at a density of 5000 cells/well and incubated in a 37°C, 5% CO2 incubator for 24 h to allow cell adhesion. The experiment was divided into two parts:

①Assessment of Puerarin's Effects on Cell Viability:

- The α‐MEM culture medium was replaced with varying concentrations of Puerarin (0, 0.01, 0.1, 1, 10, and 100 μmol/L) for 24 and 48 h.
- Following incubation, 10 μL of CCK‐8 solution was added to each well and incubated for 2 h.
- Optical density (OD) values were measured at 450 nm using a multifunctional microplate reader.

②Evaluation of Puerarin's Impact on NPC Under LPS Stimulation:

- Cells were stimulated with LPS at a concentration of 100 ng/mL for 24 h.^37^
- Subsequently, the medium was replaced with MEM/F12 containing various concentrations of puerarin (Pue) and cells were cultured for an additional 24 h.
- CCK8 detection was performed to assess the cell viability.

##### Western blot

The NPCs were pipetted and evenly distributed into a 6‐well plate at a density of 2 × 10^5 cells/well, and divided into three groups: the control group, the model group (LPS group), and the optimal concentration group determined by CCK‐8 (LPS + Pue Group). The model group was treated with LPS for 24 hours, followed by replacement with MEM/F12 medium. The LPS + Pue group received LPS intervention for 24 hours, followed by treatment with the optimal concentration of Pue for an additional 24 hours. The control group was cultured with MEM/F12 medium throughout the entire process. After the intervention, cells were washed three times with PBS, and RIPA lysis buffer was added to extract proteins from the cells. The proteins were separated by SDS‐PAGE, transferred to a PVDF membrane, and incubated with corresponding primary and secondary antibodies for detection. Primary antibodies used included IL‐1β, TNF‐a, CASP 3, CASP8, and BCL2, along with their respective secondary antibodies. The developed film was analyzed using a chemiluminescence imaging system, and ImageJ software was employed to calculate the gray value of each band.

##### RNA extraction and qPCR analysis

The experiment was similarly divided into control, model, and Pue groups. Prepared cells were digested and centrifuged, and the supernatant was removed. Trizol (1 mL) was added, thoroughly mixed by pipetting, and incubated at room temperature for 5 minutes. Then, chloroform (0.2 mL) was added to the Trizol, followed by shaking and incubation at room temperature for 10 minutes. After centrifugation at 12000 rpm at 4°C for 15 minutes, the mixture separated into three layers, with RNA in the upper colorless aqueous phase. The upper layer was aspirated into a new enzyme‐free centrifuge tube. The same volume of isopropyl alcohol was added, thoroughly mixed, and incubated at room temperature for 3 minutes. Subsequently, centrifugation was performed at 12000 rpm for 10 minutes at 4°C. Then, 1 mL of 75% absolute ethanol was added, followed by centrifugation at 10000 rpm for 5 minutes at 4°C. The supernatant was removed, and the remaining solution was left at room temperature for 3 minutes. Finally, 30–50 μL of enzyme‐free water was added, and RNA concentration was detected using a nanodrop. RNA (1 μg) was reverse‐transcribed into cDNA according to the manufacturer's instructions. Subsequently, qPCR experiments were performed, and normalization was conducted using β‐actin as a reference gene. The relative gene expression levels were calculated using the delta–delta CT (ΔΔCT) method. The primer sequences used in the experiment are shown in Table 1.

**TABLE 1:** The sequence of the primers used in the experiment.

Gene	Forward (5′‐3′)	Reverse (5′‐3′)
IL‐1β	TCTGTACCTGTCCTGCGTGT	ACTGGGCAGACTCAAATTCC
TNF‐α	CAGCCTCTTCTCCTTCCTGAT	GCCAGAGGGCTGATTAGAGA
BCL2	TGTCCCTTTGACCTTGTTTCT	TCATTTGCCATCTGGATTTT
Caspase3	CCTGGTTCATCCAGTCGCTT	TCTGTTGCCACCTTTCGGTT
Caspase8	ACTTTGCCAGAGCCTGAGAG	GCATCTGTTTCCCCATGTTT
β‐Actin	GCACCACACCTT CTACAATG	TGCTTGCTGATC CACATCTG

##### Flow Cytometry

The experimental grouping and intervention steps were as described above. After the intervention, the cell culture medium of each group was collected, the cells were washed once with PBS, and the collected cell culture medium was added after digestion. The supernatant was discarded after centrifugation, and the cells were resuspended with pre‐cooled PBS, centrifuged at 1000 g for 5 minutes, and the supernatant was discarded. Then, the cells were treated according to the instructions of the apoptosis detection kit, and finally analyzed using a flow cytometer. Flowjo software was used for data analysis.

#### 2.2.3 Data Processing and Statistical Analysis

Data from each experiment were analyzed using GraphPad Prism (version 9.5). Normality of the data was assessed using the Shapiro–Wilk test. For data that followed a normal distribution, comparisons between two groups were conducted using t‐test, while one‐way ANOVA followed by post‐hoc test was used for comparisons among multiple groups. For non‐normally distributed data, the non‐parametric test (Mann–Whitney U test) was applied. All quantitative data are reported as mean ± standard deviation (SD). Significance was defined as a p‐value <0.05. Illustrations and figures were generated using GraphPad Prism and Adobe Illustrator (version 2023).

## 3 RESULTS

### 3.1 Bioinformatics data results

#### 3.1.1 DGA results

**FIGURE 1:**
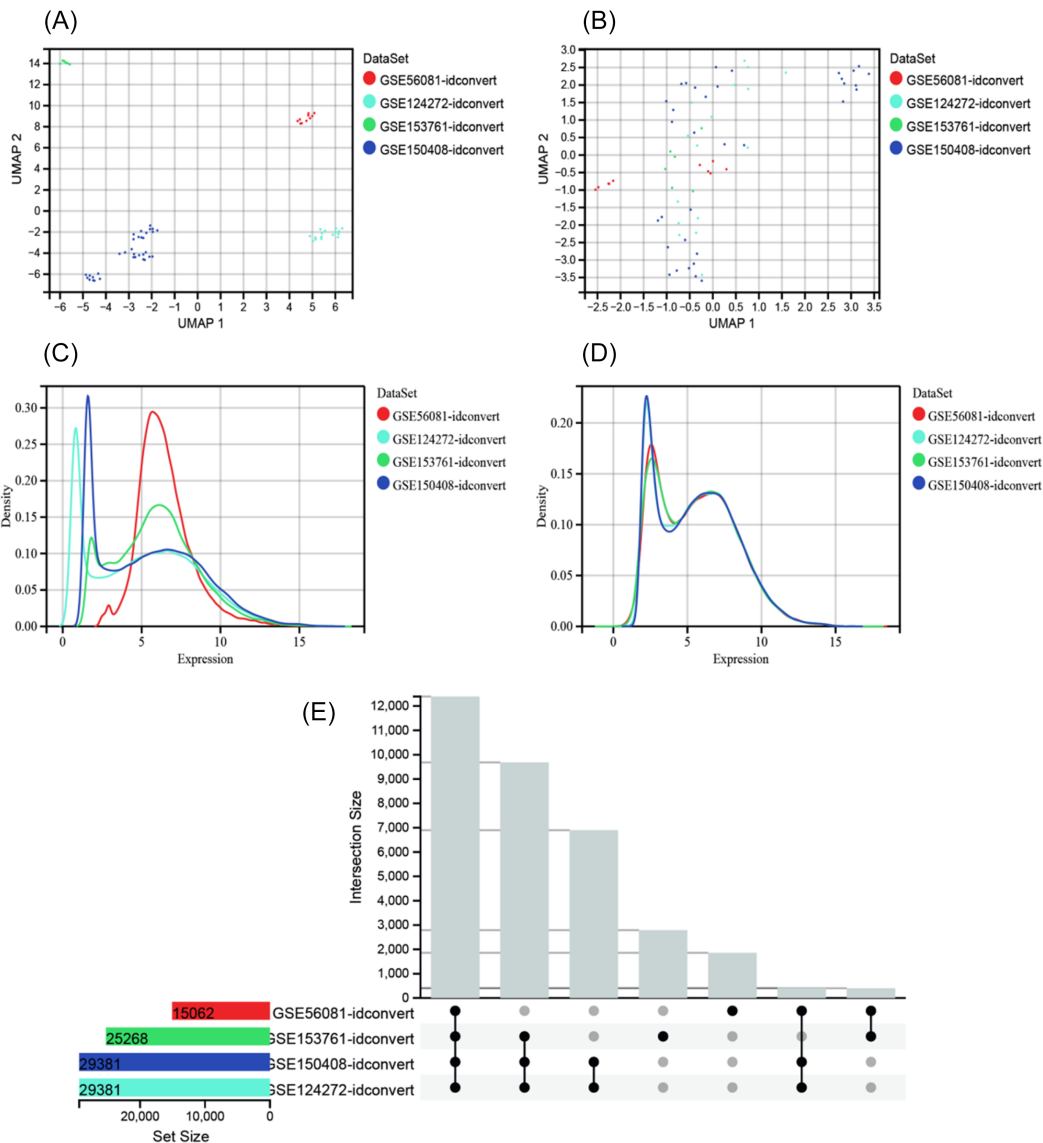
(A–D) Batch effect removal diagram: (A). The samples of each data set are clustered together before the batch effect is removed, indicating that there is a batch effect. (B). After the batch effect is removed, between the various data sets. The samples are clustered and intertwined with each other, indicating that the batch effect is better removed. (C). Before removing the batch effect, the sample distribution of each data set is quite different, indicating that there is a batch effect; (E). After removing the batch effect, the data distribution between each data set tends to be consistent, and the mean and variance are similar. EIntersection summary diagram of different sample data.

**FIGURE 2:**
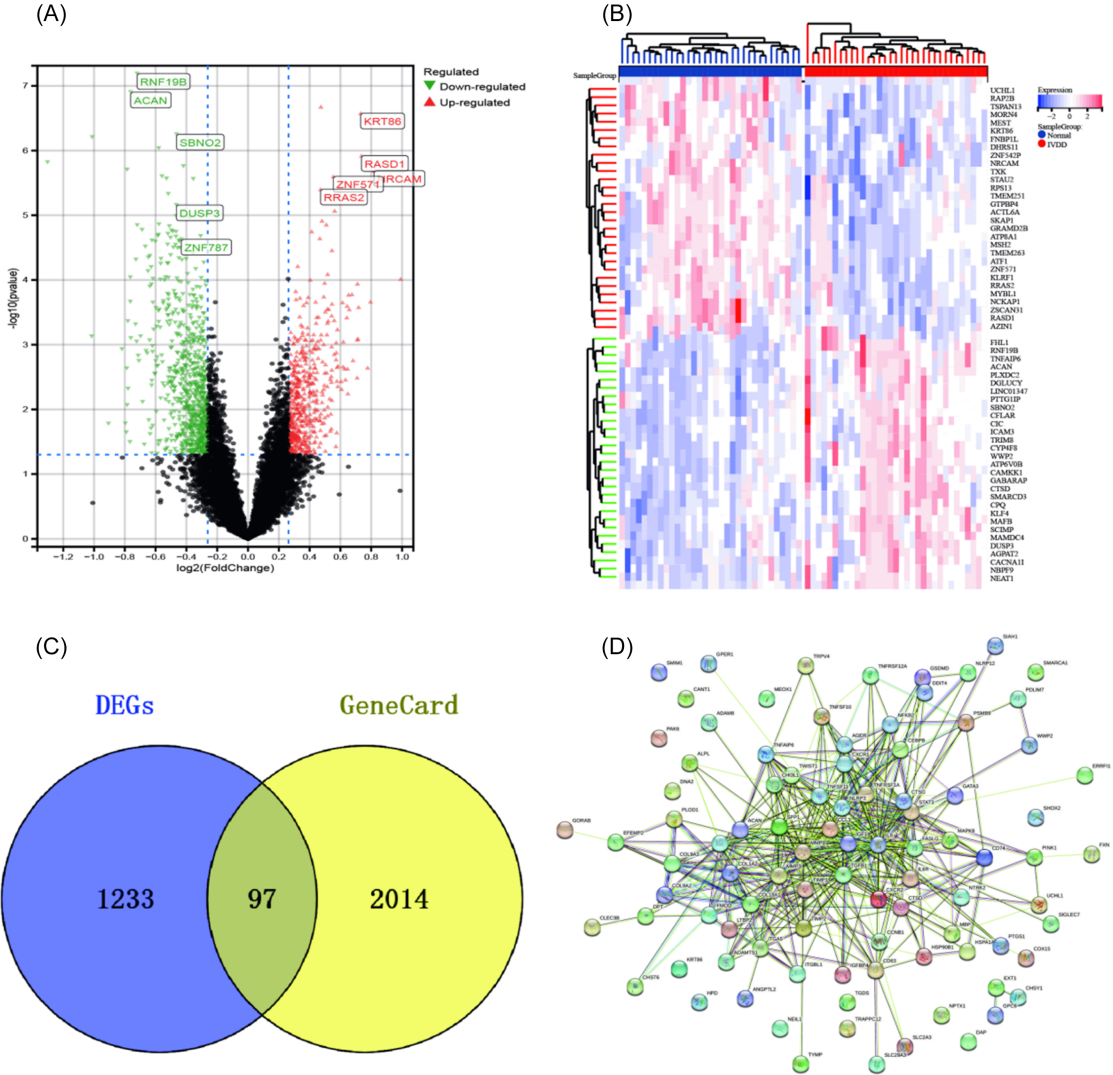
Differential gene analysis (DGA): (A). Difference volcano plot based on effective matrix original data; (B). Difference heat map based on effective matrix original data; (C). Intersection diagram of differential genes and disease data; (D). Protein interaction network diagram of intersection targets.

The GSE56081, GSE124272, GSE153761, and GSE150408 datasets were downloaded from the GEO database. After excluding post‐treatment samples from the GSE150408 dataset, a total of 66 samples were included, comprising 33 IVDD samples and 33 normal samples. Upon ID transformation, data fusion, and batch effect removal, the effective matrix raw data containing gene names was obtained (Figure 1, Supplementary Table S2). Utilizing the effective matrix raw data, the “limma” tool was employed for differential expression analysis of genes. The volcano plot illustrating differences between IVDD samples and normal control samples revealed 1330 significantly differentially expressed genes (DEGs) out of a total of 12 391 genes, including 549 up‐regulated genes and 781 down‐regulated genes (Figure 2A, Supplementary Table S3). Among these, the top 5 up‐regulated genes include KRT86, RASD1, NRCAM, ZNF571, and RRAS2, with log(fold‐change) (logFC) values of 0.73, 0.74, 0.82, 0.55, and 0.47, respectively (all p‐values <0.01, values rounded to two decimal places). The top 5 down‐regulated genes include RNF19B, ACAN, SBNO2, DUSP3, and ZNF787, with logFC values of −0.72, −0.76, −0.46, −0.47, and − 0.43, respectively (all p‐values <0.01, values rounded to two decimal places). Furthermore, a differential heatmap was generated to depict the difference levels of the top‐ranked differential genes between the normal and IVDD groups (Figure 2B). Through the GeneCard database, a list of 2111 disease‐related targets for IVDD was obtained (Supplementary Table S4). By intersecting the significantly different genes with the GeneCard disease data, 97 intersection genes were identified (Figure 2C). These genes represent potent disease‐related targets associated with IVDD. Constructing a PPI network using the STING tool revealed interactions among these 97 intersection genes (Supplementary Table S5, Figure 2D).

#### 3.1.2 WGCNA results

**FIGURE 3:**
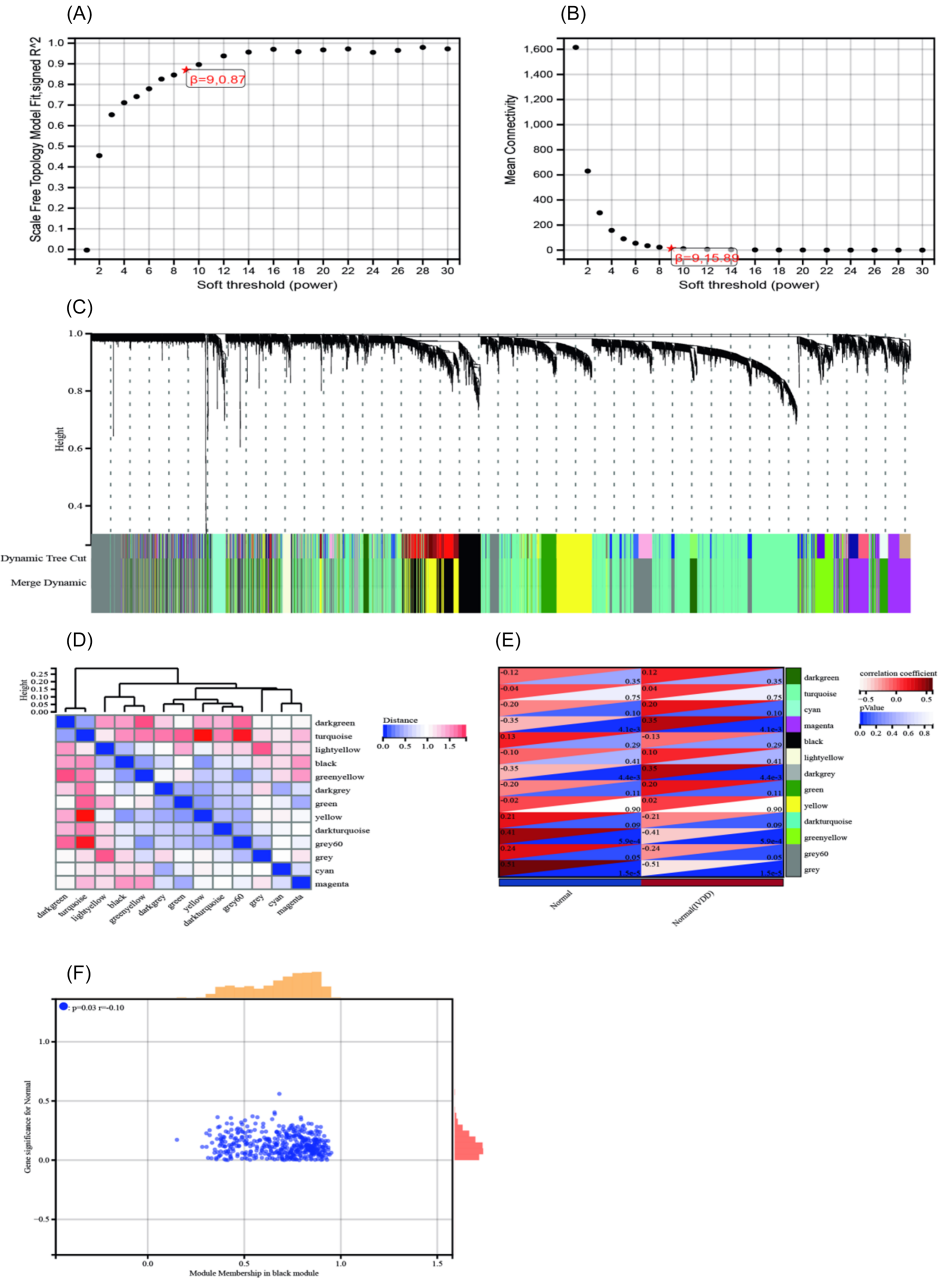
Results of WGCNA: (A). The corresponding scale‐free topological model fits the fitting index under different soft threshold powers; (B). The corresponding average connectivity values under different soft threshold powers; (C). Cluster tree shape of genes Figure; (D). Cluster analysis of module feature vectors of each module; (E). Correlation analysis of modules with clinical characteristics; (F). Correlation analysis between MM and GS.

Samples that did not cluster with the main group were considered outliers, and genes with expression levels below the threshold in 50% of the samples were considered low‐expression genes. After excluding abnormal samples and filtering genes, the expression profiles of 12 391 genes were extracted from the four datasets and utilized to construct a weighted gene co‐expression network. Setting the soft threshold power to 9 resulted in a scale independence of 0.87 and an average connectivity of 15.89 (Figure 3A,B). By setting the cutting height to 0.25 and the minimum module size to 30, 13 distinct co‐expression modules were obtained through dynamic tree cutting (Figure 3C). Cluster analysis of module feature vectors for each module revealed that turquoise and gray60, as well as turquoise and yellow modules, exhibited the largest distance (Figure 3D). Subsequently, a correlation analysis between each module and clinical characteristics was conducted. The turquoise module showed a positive correlation with IVDD (correlation coefficient = 0.04, *p* = 0.75), while the gray60 module displayed a negative correlation with IVDD (correlation coefficient = −0.24, *p* = 0.5), and the yellow module exhibited a positive correlation with IVDD (correlation coefficient = 0.02, *p* = 0.90) (Figure 3E). Additionally, correlation analysis between module membership (MM) and gene significance (GS) indicated a strong correlation between these genes, modules, and phenotypes (r = −0.10, *p* = 0.03) (Figure 3F). Finally, based on the classification of each module, a total of 1176 module genes were extracted, with the turquoise module yielding the highest number of extracted genes at 378 (Supplementary Table S6).

#### 3.1.3 Hub genes

**FIGURE 4:**
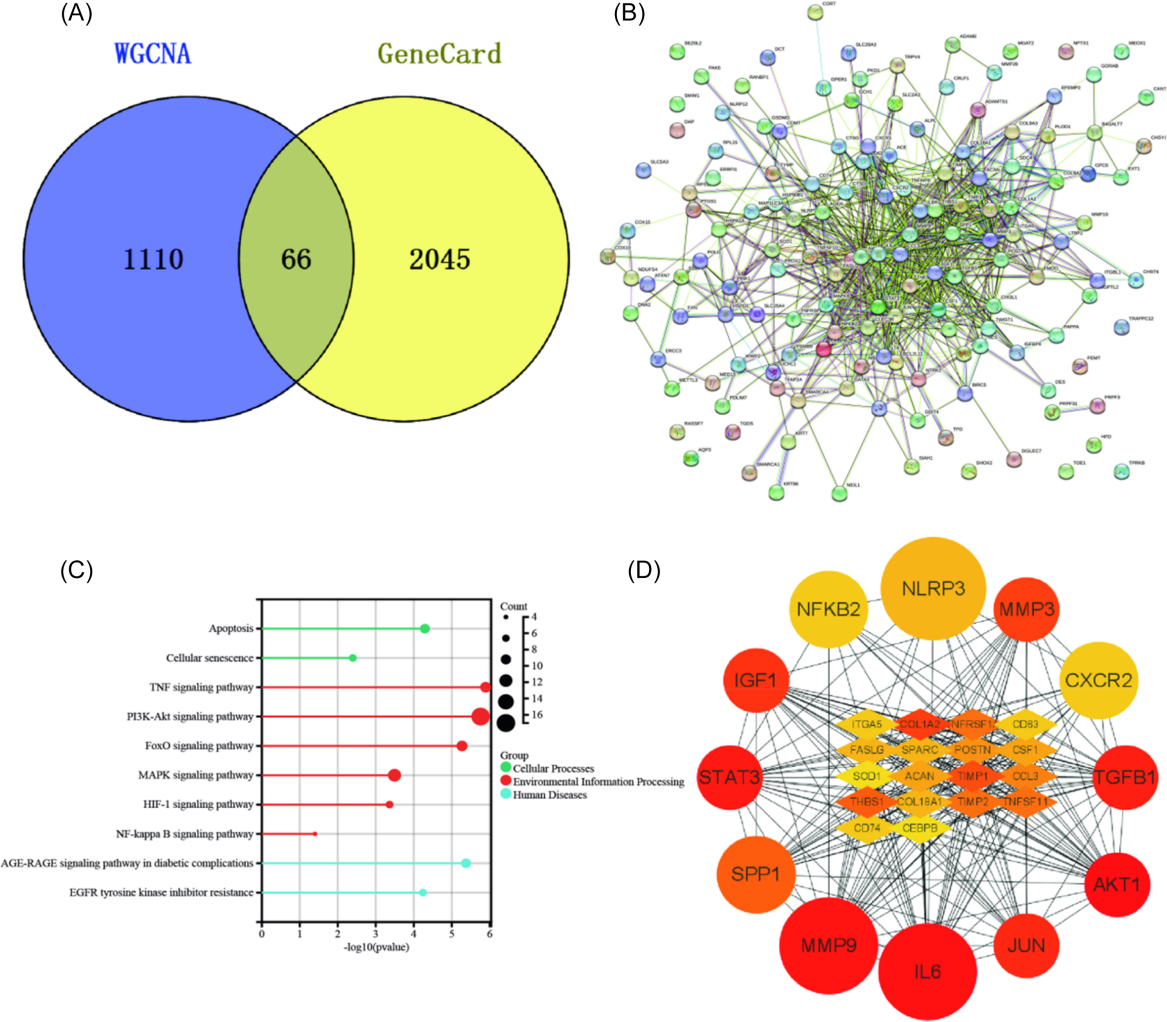
Intersection gene screening: (A). WGCNA module genes and GeneCard disease data were intersected again to obtain 66 module key hub genes; (B). 66 module key hub gene protein interaction network diagram; (C). KEGG signaling pathway analysis of intersection genes; (D). Hub gene selection based on intersection genes.

WGCNA identified 1176 module genes, and their intersection with GeneCard disease data yielded 66 key hub genes of the module (Figure 4A). Combining the 97 effective disease genes with the 66 module key hub genes and removing duplicates resulted in 158 hub genes effectively regulating IVDD for subsequent analysis (Figure 4B, Supplementary Table S5). Subsequent analysis identified the top 30 important regulatory hub genes (Figure 4C). Differential expression analysis revealed that among the 30 hub genes, NLRP3, AKT1, IL6, MMP9, and TGFB1, among others, may play crucial roles in IVDD (Supplementary Table S7). Further analysis was conducted to examine how the 158 hub regulatory genes participate in IVDD through KEGG signaling pathway analysis. This analysis revealed enrichment in a total of 211 signaling pathways (Supplementary Table S8). Following comprehensive sorting based on P value and Count count, Cellular Processes mainly involved Apoptosis and Cellular Senescence signaling pathways, while Environmental Information Processing mainly included the TNF signaling pathway, PI3K‐Akt signaling pathway, FoxO signaling pathway, MAPK signaling pathway, HIF‐1 signaling pathway, and NF‐kappa B signaling pathway. Human Diseases primarily encompassed the AGE‐RAGE signaling pathway in diabetic complications and EGFR tyrosine kinase inhibitor resistance (Figure 4D).

### 3.2 Molecular mechanism of puerarin in treating IVDD

**FIGURE 5:**
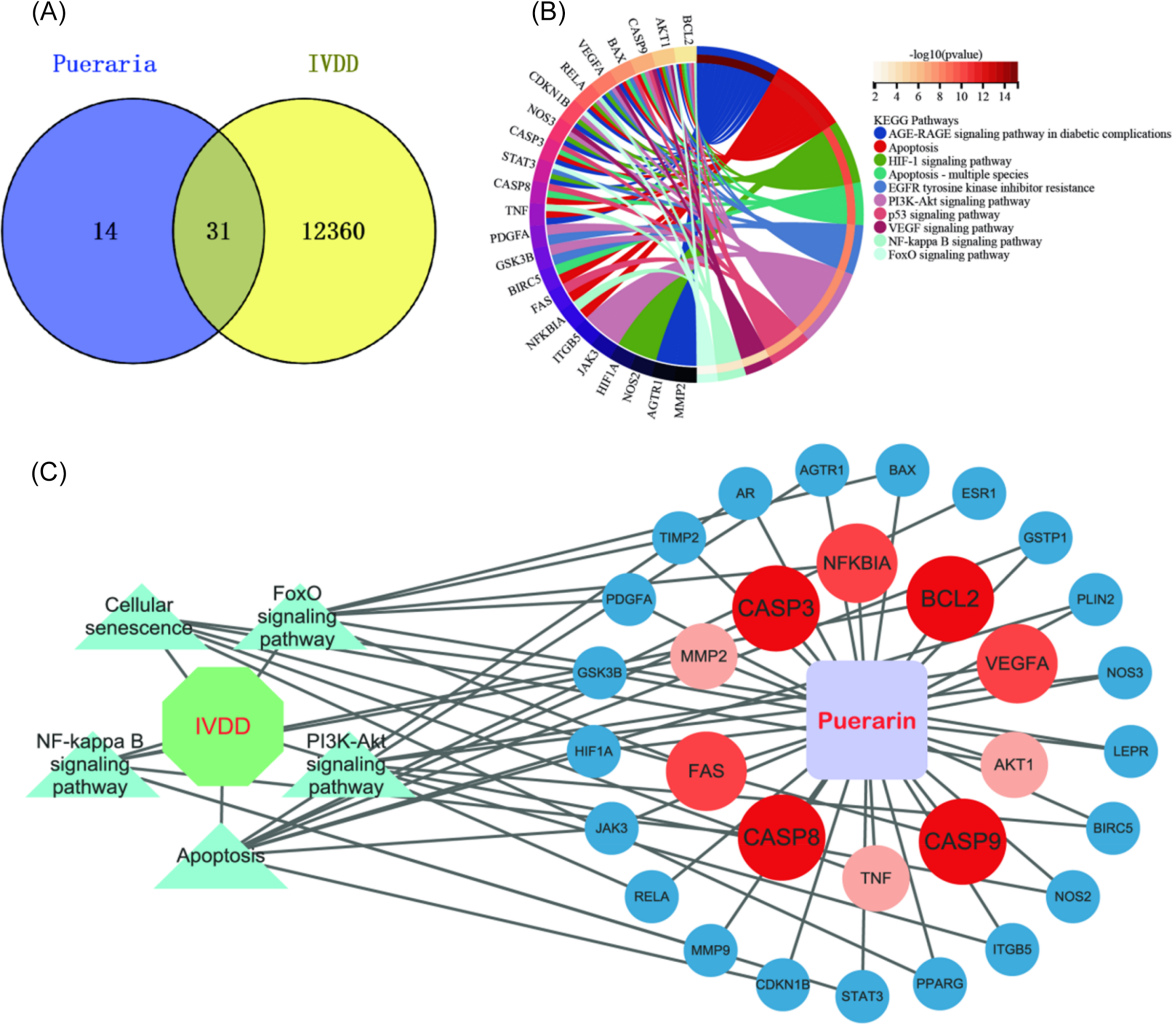
(A). Intersecting targets of puerarin in treating IVDD; (B). KEGG enrichment analysis of therapeutic targets; (C). Network regulation diagram of puerarin in treating IVDD.

After screening through TCMSP, we identified 44 effective targets of puerarin. The intersection of these drug action targets with the 12 391 original matrix effective targets yielded 31 potential therapeutic targets (Figure 5A, Supplementary Table S9). KEGG enrichment analysis based on these 31 targets revealed that puerarin treatment of IVDD involves 180 signaling pathways, with a focus on apoptosis, PI3K‐Akt signaling pathway, FoxO signaling pathway, HIF‐1 signaling pathway, and NF‐kappa B signaling pathway, among others (Figure 5B, Supplementary Table S10). Furthermore, the network regulatory map illustrated the inclusion of key apoptosis hub genes, suggesting that puerarin may treat IVDD through the apoptosis pathway and related targets (Figure 5C). Significant differences were observed in the expression of these hub genes in IVDD.

#### 3.2.1 Results of molecular docking

**FIGURE 6:**
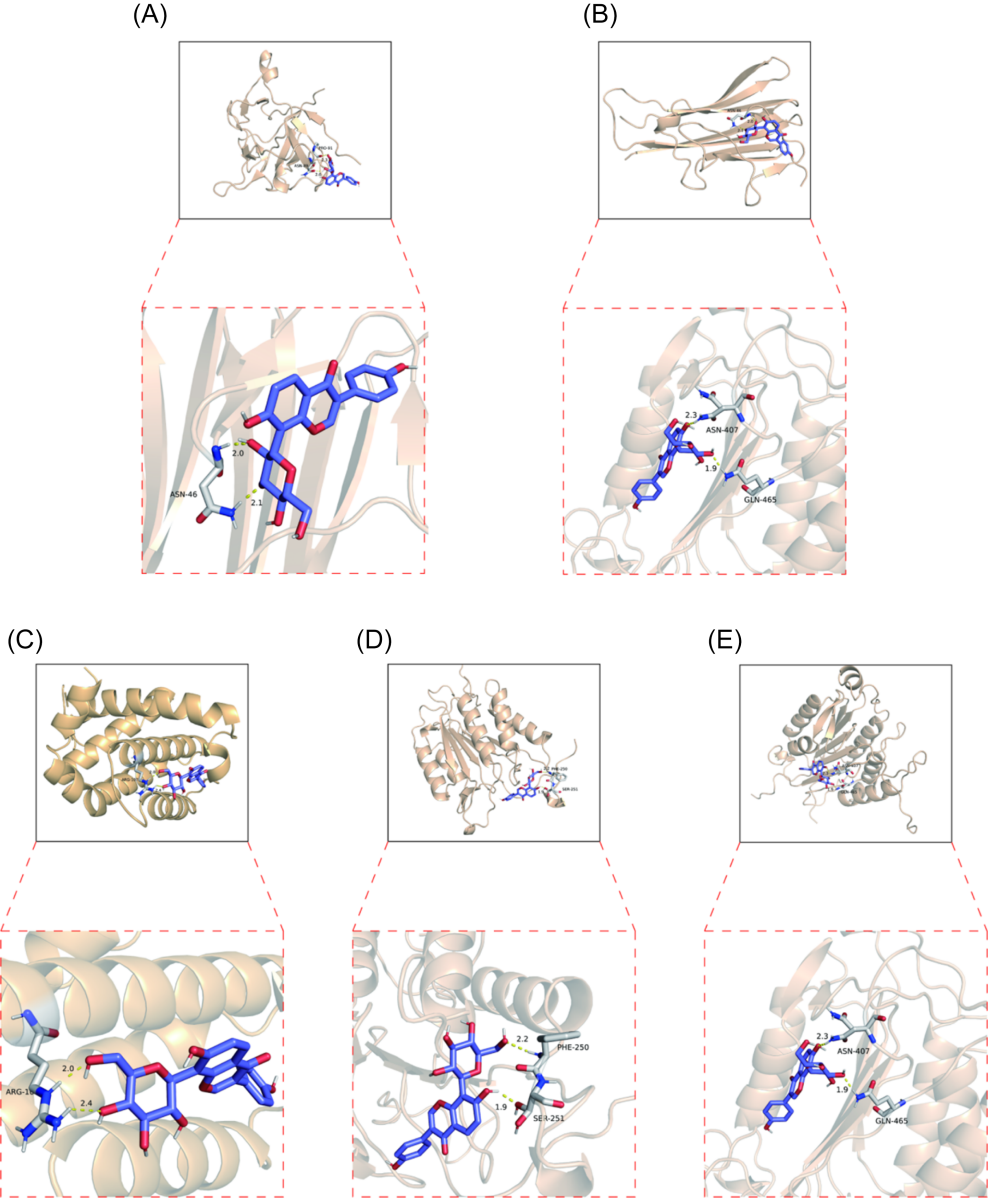
Molecular docking diagrams of puerarin with IL‐1β, TNF‐α, BCL2, Caspase3, and Caspase8. (A). Puerarin‐IL‐1β; (B). Puerarin‐IL‐1β; (C). Puerarin‐BCL2; (D). Puerarin‐Caspase3; D. Puerarin‐Caspase8.

In this study, molecular docking was conducted to assess the binding activity of puerarin with key targets. A binding energy of ≤ − 5 kcal/mol is typically indicative of favorable binding properties. The binding energies are presented in Table 2, while the schematic diagram of molecular docking is depicted in Figure 6.

**TABLE 2:** Binding abilities of puerarin with IL‐1β, TNF‐α, BCL2, Caspase3, and Caspase8, respectively.

Molecule name	Binding energy(kcal/mol)
IL‐1β	−5.5
TNF‐α	−5.8
BCL2	−6.9
Caspase3	−7.6
Caspase8	−7.0

##### Puerarin promotes NPC proliferation

**FIGURE 7:**
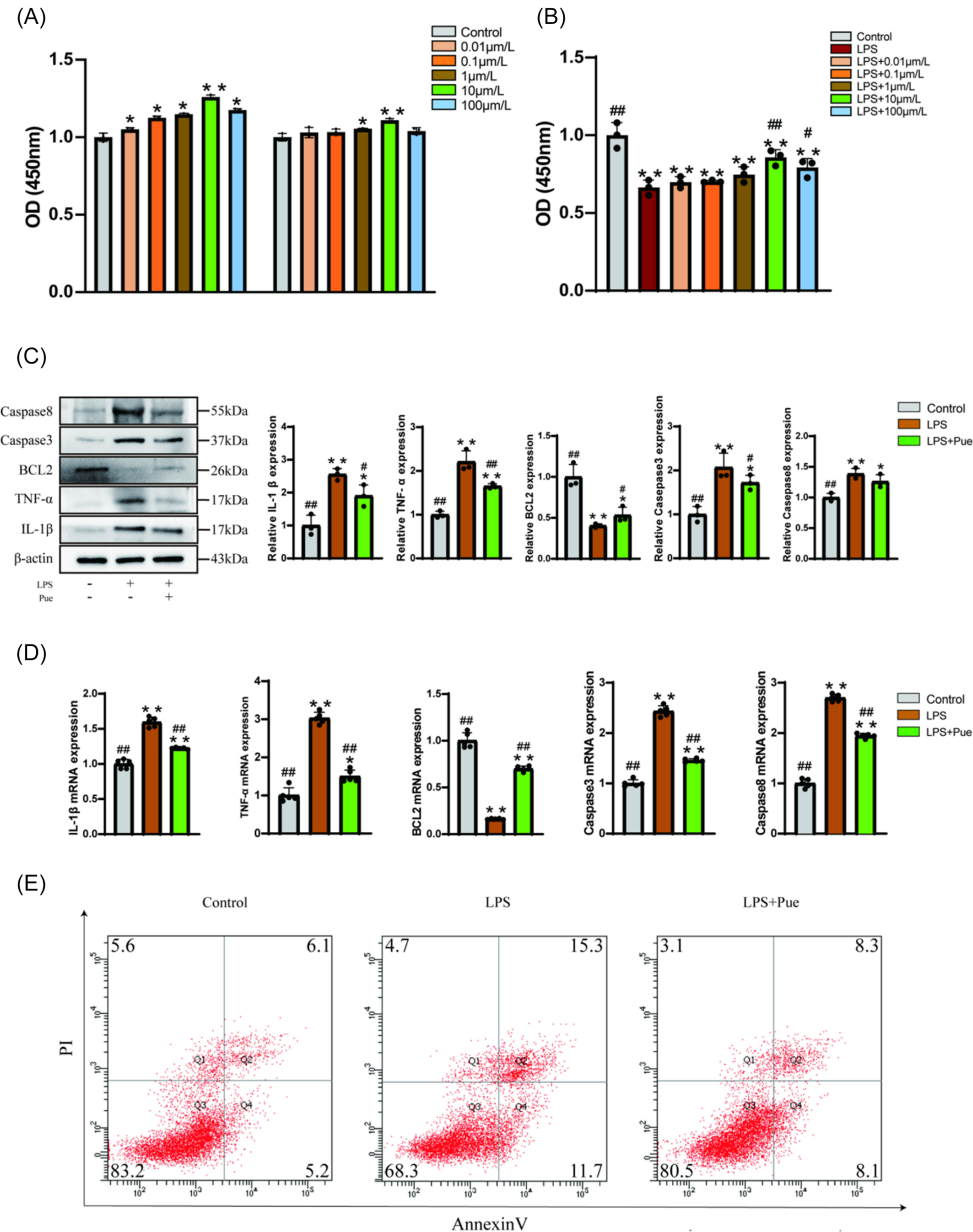
(A). Effects of different concentrations of puerarin on NPC proliferation after 24 h and 28 h intervention; (B). Impact of varied puerarin concentrations on NPC proliferation following 24 hours of LPS stimulation; (C). Protein expression levels of IL‐1β, TNF‐α, BCL2, Caspase3, and Caspase8 in each group (each experiment was conducted with 3 technical replicates); (D). Relative mRNA expression levels of IL‐1β, TNF‐α, BCL2, Caspase3, and Caspase8 in each group respectively (delta‐CT; each experiment was performed with 4 technical replicates); (E). Flow cytometry results of apoptosis of nucleus pulposus cells (NPCs) in each group (The values in the four corners represent the percentage of each quadrant). (Bars represent the mean values of the measurements, and error bars represent the standard deviation (SD) of the mean; Compared to the control group, *p < 0.05, **p < 0.01; compared to the Pue group, ^#^ *p* < 0.05, ^##^ *p* < 0.01).

To assess the impact of puerarin on NPC proliferation, we directly treated the cells with varying concentrations of puerarin. Results indicated that after 24 hours of intervention, NPC proliferation significantly increased compared to the control group across all puerarin concentrations (*p* < 0.05), with the most pronounced effect observed at 10 μmol/L (*p* < 0.01). Following 48 hours of intervention, only 1 and 10 μmol/L puerarin demonstrated a significant promotion of NP cell proliferation compared to the control group (*p* < 0.05), with 10 μmol/L exhibiting the most notable effect. Interestingly, the proliferative effect of puerarin diminished after 48 hours of intervention compared to the 24‐hour time point. These findings suggest that puerarin can enhance NPC proliferation at appropriate concentrations and intervention durations (Figure 7A). To further validate the impact of puerarin on NPC proliferation under LPS intervention, we first treated the cells with LPS followed by puerarin intervention. Results depicted in Figure 7B indicate that LPS intervention significantly inhibited NPC proliferation (*p* < 0.01). However, treatment with puerarin at each concentration effectively counteracted the inhibitory effect of LPS (*p* < 0.01), with 10 μmol/L exhibiting the most pronounced rescue effect. Collectively, these findings demonstrate that puerarin can effectively promote NPC proliferation.

##### Puerarin promotes NPC proliferation by inhibiting apoptosis

Based on our bioinformatics analysis, apoptosis emerges as a crucial pathway in puerarin‐mediated IVDD treatment, with NPCs playing a pivotal role in disease regulation. We speculate that puerarin regulates key apoptosis targets such as IL‐1β, TNF‐a, CASP 3, CASP8, and BCL2 to enhance NPC proliferation. As depicted in Figure 7C, Western blot analysis revealed increased expression of IL‐1β, TNF‐a, CASP 3, and CASP8, along with decreased BCL2 expression in the model group compared to the control group (*p* < 0.05), indicating LPS‐induced apoptosis in NPCs. However, intervention with puerarin resulted in reduced expression of IL‐1β, TNF‐a, CASP 3, and CASP8, coupled with increased BCL2 expression (*p* < 0.05), indicating puerarin's inhibitory effect on LPS‐induced apoptosis in NPCs. Consistent findings were observed in PCR‐based RNA level analysis (Figure 7D), while flow cytometry analysis further confirmed that puerarin significantly attenuated LPS‐induced NPC apoptosis (Figure 7E). Collectively, these results underscore puerarin's ability to inhibit NPC apoptosis and thereby promote their proliferation.

## 4 DISCUSSION

LBP affects more than 84% of people worldwide, with IVDD being a major underlying cause. However, the exact pathological mechanisms of IVDD remain incompletely understood.^38^ Current evidence suggests a strong association between NPC death, immune inflammation, and various IVDD processes.^7^ Specifically, inflammatory cytokines like IL‐1β and TNF‐α, along with apoptosis‐related pathways involving CASP 3, CASP8, and BCL2, are implicated in IVDD progression.^22^ While significant strides have been made in understanding these pathways, further elucidation of their molecular mechanisms is warranted. In our study, we aim to delve into the mechanisms underlying apoptosis and inflammation in IVDD, offering insights for clinical diagnosis and treatment. Puerarin, a traditional Chinese medicine, has shown potential in alleviating symptoms of LBP associated with IVDD in preclinical and some clinical studies. Its protective effects are thought to involve the downregulation of Fas expression and upregulation of Bcl‐2 expression in intervertebral disc tissue. However, further research is needed to fully understand its therapeutic role and validate its clinical efficacy in diverse patient populations.^27^ Building on this knowledge, our study seeks to provide a deeper understanding of the molecular mechanisms through which puerarin regulates apoptosis in IVDD.^39^

In this study, we initially conducted a comprehensive analysis of gene expression profiles obtained from IVDD patients and healthy individuals using the GEO database. Through this analysis, we identified 97 differentially expressed hub genes. Subsequently, employing a combination of PPI network and WGCNA, we further pinpointed 30 hub genes associated with cell senescence, immune inflammation, and apoptosis regulation. Notably, key genes such as NLRP3, AKT1, IL6, MMP9, and TGFB1 were identified as pivotal players in these processes. While apoptosis is considered the primary cell death pathway in NP cells, strategies aimed at inhibiting apoptosis in IVDD treatment have yielded incomplete results, highlighting the need for a deeper exploration of its molecular mechanisms.^8^ Extensive research has demonstrated that dysregulated apoptosis can lead to impaired tissue repair and contribute to various inflammatory diseases, including arthritis, sepsis, cancer, atherosclerosis, and Parkinson's disease.^40^ Although previous studies have recognized apoptosis as a crucial regulatory process in IVDD, the efficacy of alleviating IVDD by targeting NPC apoptosis remains inconclusive. Hence, our study seeks to shed further light on the molecular mechanisms underlying NPC apoptosis in IVDD, aiming to provide valuable insights for the development of more effective therapeutic interventions.

Through bioinformatics analysis, we have identified that IVDD primarily involves dysregulation of cell apoptosis, cell senescence, and key signaling pathways such as PI3K‐Akt, FoxO, and NF‐kappa B. Central to these processes are inflammatory factors and regulatory targets including NLRP3, AKT1, IL6, MMP9, TGFB1, IL‐1β, TNF‐a, CASP 3, CASP8, and BCL2. Apoptosis is recognized as a contributing factor in IVDD development, often triggered by various stimuli through different pathways, including death receptors, endoplasmic reticulum stress, and mitochondrial dysfunction.^19^, ^41^ Mitochondrial dysfunction, characterized by oxidative stress‐induced alterations in Bcl‐2/Bax balance, can activate the caspase family (such as CASP 3 and CASP8), ultimately leading to apoptosis.^42^ The caspase family, crucial in apoptotic signaling, not only links cell apoptosis with inflammatory responses but also regulates the expression of key inflammatory factors like IL‐1β and TNF‐a, thus influencing IVDD progression.^43^ However, direct targeting of caspases may pose challenges due to their downstream location and susceptibility to various regulatory signals. Therefore, indirect modulation of degenerative intervertebral discs might not achieve desired therapeutic outcomes consistently.^44^ In our study, we observed that puerarin treatment significantly reduced the levels of IL‐1β, TNF‐a, CASP 3, and CASP8 in LPS‐induced NPCs while increasing Bcl‐2 expression. These findings suggest that puerarin effectively mitigates inflammatory responses and apoptotic signaling in NPCs, potentially offering therapeutic benefits for IVDD.

In addition to apoptosis, our study has identified the involvement of cell senescence, the PI3K‐Akt signaling pathway, the FoxO signaling pathway, and the NF‐kappa B signaling pathway in IVDD.^7^ Research suggests that cellular senescence markers are elevated in Casp‐3 apoptosis gene knockout mice, particularly in older mice. Interestingly, Casp‐3 knockout inhibits injury‐related IVDD but accelerates aging‐related IVDD,^43^ indicating a complex interplay between apoptosis and senescence in IVDD progression. However, further investigation is needed to elucidate the precise underlying mechanism. Moreover, intervertebral disc aging is associated with increased production of reactive oxygen species (ROS). ROS, generated during aerobic metabolism, accumulate in cells under stress conditions, leading to oxidative damage to proteins, DNA, and lipids.^45^ Elevated levels of ROS, often observed in inflammatory tissues due to immune cell activation, contribute to excessive inflammation. IVDD, characterized by local increases in IL‐1β and TNF‐a, may induce apoptosis through mitochondrial dysfunction and endoplasmic reticulum stress, further exacerbating oxidative stress.^46^ IVDD, characterized by local increases in IL‐1β and TNF‐a, may induce apoptosis through mitochondrial dysfunction and endoplasmic reticulum stress, further exacerbating oxidative stress.^46^ ROS activation triggers various signaling pathways in intervertebral cells, including the PI3K‐Akt, FoxO, and NF‐kappa B pathways. NF‐κB, a transcription factor regulating pro‐inflammatory cytokine genes, represents a potential therapeutic target for inflammatory diseases.^47^

However, while our study has demonstrated that NP cell apoptosis is a key process in IVDD pathogenesis and that puerarin can inhibit this apoptosis by regulating key targets such as IL‐1β, TNF‐a, CASP 3, CASP8, and BCL2, several limitations must be acknowledged. Firstly, our in vitro model using LPS to induce degeneration may not fully replicate the complex in vivo environment of IVDD, as the multifactorial nature of the condition involves various biological, mechanical, and biochemical factors. Additionally, we did not consider the use of apoptosis inhibitors, which could potentially influence the observed effects of puerarin. Furthermore, the use of primary NPCs from a single donor may impact the generalizability of our results, as findings could be specific to that donor. Future research should incorporate multiple donors to validate the reproducibility of our observations. While we have explored various signaling pathways, the precise molecular mechanisms through which puerarin influences cell senescence and oxidative stress, as well as its validation in relevant pathways, require further investigation. A more extensive exploration of these aspects will be necessary to fully understand the therapeutic potential of puerarin in IVDD.

## 5 CONCLUSION

In conclusion, we have established a predictive model for IVDD based on apoptosis hub genes, including IL‐1β, TNF‐a, CASP 3, CASP8, and BCL2. Our findings highlight the pivotal role of cell apoptosis and senescence in the pathogenesis of IVDD, along with dysregulated signaling pathways such as PI3K‐Akt, FoxO, and NF‐kappa B pathways. Importantly, our study confirms that puerarin effectively treats IVDD by attenuating the production of pro‐inflammatory factors within the intervertebral disc and inhibiting apoptosis of NPCs.

## AUTHOR CONTRIBUTIONS

Xiaoqiang Wang: Writing—original draft. Chao Song: Writing—original draft. Daqian Zhou: Formal analysis, Methodology. Yongliang Mei: Visualization, Weiye Cai: Supervision, Rui Chen: Methodology, Jiale Lv and Houyin Shi: Conceptualization, Writing—review and editing. Zongchao Liu: Conceptualization, Funding acquisition. All authors listed have made a substantial contribution to the work.

## FUNDING INFORMATION

This research was supported by Luzhou's major scientific and technology research and development project (Nos.2022‐SYF‐42), Luzhou Science and Technology Program‐Innovation Seedlings (Nos.2022‐RCM‐178), Luzhou Municipal People's Government‐Southwest Medical University Science and Technology Strategic Cooperation Climbing Plan Project (Nos.2021LZXNYD‐D02), Joint Innovation Special of the Sichuan Provincial Science and Technology Plan (Nos.2022YFS0609 / 2022YFS0609‐B3), the Sichuan Science and Technology Department Project Development Project (Nos.2022YFS0391/22ZDYF0512), the Program for Special project of Traditional Chinese Medicine scientific research of Sichuan Science and Traditional Chinese Medicine Administration (Nos. 2023MS019).

## CONFLICT OF INTEREST STATEMENT

The authors have no conflicts of interest to disclose.

## Supporting information


**Supplementary Table S1:** Information of donors in each database.

**Supplementary Table S2.** Original matrix data after removing batch effects.
**Supplementary Table S3.** Differentially expressed genes(DEGs).
**Supplementary Table S4.** Targets of IVDD in GeneCard.
**Supplementary Table S5.** The intersection of genes.
**Supplementary Table S6.** Module hub genes obtained by WGCN analysis.
**Supplementary Table S7.** Hub genes screened using Cytoscope.
**Supplementary Table S8.** KEGG signaling pathway analysis based on 158 hub genes.
**Supplementary Table S9.** Targets of puerarin.
**Supplementary Table S10.** KEGG pathway analysis based on 31 hub genes for puerarin treatment of IVDD.

## Data Availability

All relevant data are within the manuscript and its additional files.
