# Supplementary material for: Exploring the therapeutic potential of puerarin on intervertebral disc degeneration by regulating apoptosis of nucleus pulposus cells
Source: JOR Spine. 2024 Dec 11;7(4):e70020. doi: 10.1002/jsp2.70020 (PMC11632247; doi:10.1002/jsp2.70020)
Supplement: Supplementary file 1 — Supplementary Table S1: Information of donors in each database. [file JSP2-7-e70020-s001.docx]

Supplementary Table S1: Information of donors in each database.

| Sample ID | Gender | Age | Type of IVDD | Pfirrmann grade | Spinal levels |
| --- | --- | --- | --- | --- | --- |
| GSE56081 |  |  |  |  |  |
| GSM1354764 | Male | 33 | Healthy | I | L3-L4 |
| GSM1354765 | Male | 35 | Healthy | I | L3-L4 |
| GSM1354766 | Male | 41 | Healthy | I | L4-L5 |
| GSM1354767 | Female | 43 | Healthy | I | L5-S1 |
| GSM1354768 | Male | 52 | Healthy | I | L4-L5 |
| GSM1354769 | Female | 32 | LDH | Ⅴ | L4-L5 |
| GSM1354770 | Male | 38 | LDH | Ⅴ | L3-L4 |
| GSM1354771 | Male | 42 | LDH | Ⅳ | L3-L4 |
| GSM1354772 | Male | 45 | LDH | Ⅴ | L5-S1 |
| GSM1354773 | Female | 27 | LDH | Ⅳ | L4-L5 |
|  |  |  |  |  |  |
| GSE153761 |  |  |  |  |  |
| GSM4653870 | Male | 70 | IVDD | NA | NA |
| GSM4653871 | Female | 49 | IVDD | NA | NA |
| GSM4653872 | Male | 50 | IVDD | NA | NA |
| GSM4653873 | Male | 62 | Healthy | NA | NA |
| GSM4653874 | Female | 35 | Healthy | NA | NA |
| GSM4653875 | Female | 40 | Healthy | NA | NA |
|  |  |  |  |  |  |
| GSE150408 |  |  |  |  |  |
| GSM4548691 | NA | NA | Healthy | NA | NA |
| GSM4548692 | NA | NA | Healthy | NA | NA |
| GSM4548693 | NA | NA | Healthy | NA | NA |
| GSM4548694 | NA | NA | Healthy | NA | NA |
| GSM4548695 | NA | NA | Healthy | NA | NA |
| GSM4548696 | NA | NA | Healthy | NA | NA |
| GSM4548697 | NA | NA | Healthy | NA | NA |
| GSM4548698 | NA | NA | Healthy | NA | NA |
| GSM4548699 | NA | NA | Healthy | NA | NA |
| GSM4548700 | NA | NA | Healthy | NA | NA |
| GSM4548701 | NA | NA | Healthy | NA | NA |
| GSM4548702 | NA | NA | Healthy | NA | NA |
| GSM4548703 | NA | NA | Healthy | NA | NA |
| GSM4548704 | NA | NA | Healthy | NA | NA |
| GSM4548705 | NA | NA | Healthy | NA | NA |
| GSM4548706 | NA | NA | Healthy | NA | NA |
| GSM4548707 | NA | NA | Healthy | NA | NA |
| GSM4548708 | NA | NA | IVDD | NA | NA |
| GSM4548709 | NA | NA | IVDD | NA | NA |
| GSM4548710 | NA | NA | IVDD | NA | NA |
| GSM4548711 | NA | NA | IVDD | NA | NA |
| GSM4548712 | NA | NA | IVDD | NA | NA |
| GSM4548713 | NA | NA | IVDD | NA | NA |
| GSM4548714 | NA | NA | IVDD | NA | NA |
| GSM4548715 | NA | NA | IVDD | NA | NA |
| GSM4548716 | NA | NA | IVDD | NA | NA |
| GSM4548717 | NA | NA | IVDD | NA | NA |
| GSM4548718 | NA | NA | IVDD | NA | NA |
| GSM4548719 | NA | NA | IVDD | NA | NA |
| GSM4548720 | NA | NA | IVDD | NA | NA |
| GSM4548721 | NA | NA | IVDD | NA | NA |
| GSM4548722 | NA | NA | IVDD | NA | NA |
| GSM4548723 | NA | NA | IVDD | NA | NA |
| GSM4548724 | NA | NA | IVDD | NA | NA |
|  |  |  |  |  |  |
| GSE124272 |  |  |  |  |  |
| GSM3526782 | NA | NA | IVDD | NA | NA |
| GSM3526783 | NA | NA | IVDD | NA | NA |
| GSM3526784 | NA | NA | IVDD | NA | NA |
| GSM3526785 | NA | NA | IVDD | NA | NA |
| GSM3526786 | NA | NA | IVDD | NA | NA |
| GSM3526787 | NA | NA | IVDD | NA | NA |
| GSM3526788 | NA | NA | IVDD | NA | NA |
| GSM3526789 | NA | NA | IVDD | NA | NA |
| GSM3526790 | NA | NA | Healthy | NA | NA |
| GSM3526791 | NA | NA | Healthy | NA | NA |
| GSM3526792 | NA | NA | Healthy | NA | NA |
| GSM3526793 | NA | NA | Healthy | NA | NA |
| GSM3526794 | NA | NA | Healthy | NA | NA |
| GSM3526795 | NA | NA | Healthy | NA | NA |
| GSM3526796 | NA | NA | Healthy | NA | NA |
| GSM3526797 | NA | NA | Healthy | NA | NA |

Abbreviations: LDH, lumbar disc herniation; NA, not applicable (not specified in the original data).
